# Six Multiplex TaqMan^TM^-qPCR Assays for Quantitative Diagnostics of *Pseudomonas* Species Causative of Bacterial Blotch Diseases of Mushrooms

**DOI:** 10.3389/fmicb.2020.00989

**Published:** 2020-05-25

**Authors:** Tanvi Taparia, Marjon Krijger, Jennifer Hodgetts, Marc Hendriks, John G. Elphinstone, Jan van der Wolf

**Affiliations:** ^1^Biointeractions and Plant Health, Wageningen University and Research, Wageningen, Netherlands; ^2^Department of Microbial Ecology, Netherlands Institute of Ecology, Wageningen, Netherlands; ^3^Department of Plant Protection, Fera Science Limited, York, United Kingdom

**Keywords:** molecular diagnostics, “*Pseudomonas gingeri*, ” *Pseudomonas tolaasii*, *Pseudomonas edaphica*, *Pseudomonas salomonii*, soil-borne pathogens, *Agaricus bisporus*, button mushrooms

## Abstract

Bacterial blotch is a group of economically important diseases of the common button mushroom (*Agaricus bisporus*). Once the pathogens are introduced to a farm, mesophilic growing conditions (that are optimum for mushroom production) result in severe and widespread secondary infections. Efficient, timely and quantitative detection of the pathogens is hence critical for the design of localized control strategies and prediction of disease risk. This study describes the development of real-time TaqMan^TM^ assays that allow molecular diagnosis of three currently prevalent bacterial blotch pathogens: “*Pseudomonas gingeri,” Pseudomonas tolaasii* and (as yet uncharacterized) *Pseudomonas* strains (belonging to *Pseudomonas salomonii and Pseudomonas edaphica*). For each pathogen, assays targeting specific DNA markers on two different loci, were developed for primary detection and secondary verification. All six developed assays showed high diagnostic specificity and sensitivity when tested against a panel of 63 *Pseudomonas* strains and 40 other plant pathogenic bacteria. The assays demonstrated good analytical performance indicated by linearity across calibration curve (>0.95), amplification efficiency (>90%) and magnitude of amplification signal (>2.1). The limits of detection were optimized for efficient quantification in bacterial cultures, symptomatic tissue, infected casing soil and water samples from mushroom farms. Each target assay was multiplexed with two additional assays. *Xanthomonas campestris* was detected as an extraction control, to account for loss of DNA during sample processing. And the total *Pseudomonas* population was detected, to quantify the proportion of pathogenic to beneficial *Pseudomonas* in the soil. This ratio is speculated to be an indicator for blotch outbreaks. The multiplexed assays were successfully validated and applied by routine testing of diseased mushrooms, peat sources, casing soils, and water from commercial production units.

## Introduction

Cultivation of the white button mushroom (*Agaricus bisporus*), represents a global economic value of more than 4.7 billion US dollars (Sonnenberg et al., 2011). Bacterial blotch diseases render crops unmarketable by strongly affecting the esthetic quality of the caps pre-harvest and reducing the shelf life of the mushrooms post-harvest. Economic losses from this disease have been reported globally across Europe (Paine, 1919; Olivier et al., 1978), North America (Tolaas, 1915), Middle East (Bashan and Okon, 1981; Özaktan and Bora, 1994), Asia (Guleria, 1976; Chen, 1981; Suyama and Fujii, 1993; Kim et al., 1995), and Australia (Nair, 1969; Fahy, 1981). In Europe alone, blotch outbreaks can reduce the total yield up to 50% (Soler-Rivas et al., 1999). Bacterial blotch diseases of mushrooms have been well-described for over a century. They are caused by diverse fluorescent *Pseudomonas* species, probably originating from the casing soils in mushroom farms (Wong and Preece, 1980). The casing soil is a 5 cm layer of peat and lime that is placed on top of the compost, to facilitate formation of mushroom pinheads.

*P. tolaasii* is the predominant pathogen of “brown blotch,” and produces dark, sunken, brown lesions (Tolaas, 1915; Paine, 1919). It produces pitting and brown lesions on the mushroom caps by secreting the extracellular toxin “tolaasin” (Soler-Rivas et al., 1997). The biochemical mechanisms of browning, the biosynthesis of tolaasin, and its genetic regulation have been well-studied (Rainey et al., 1993; Han et al., 1994; Grewal et al., 1995). Non-pathogenic forms of *P. tolaasii*, are unable to produce this toxin. *P. tolaasii* is also a pathogen of specialty mushrooms such as *Pleurotus ostreatus, Flammulina velutipes*, and *Pleurotus eryngii* (Suyama and Fujii, 1993; González et al., 2009; Han et al., 2012).

Other *Pseudomonas* species are also known to cause brown blotch (Elphinstone and Noble, 2018; unpublished results). *P. salomonii* and *P. edaphica* strains isolated from symptomatic mushroom tissue, were recently shown to cause severe brown blotch symptoms (unpublished results). They were formerly identified as *P. tolaasii*, and taxonomically corrected in a molecular characterization of blotch-causing pseudomonads (unpublished results). This taxonomic group is closely related to two known blotch pathogens, *P. constantinii* and *P. fluorescens.* In this work, we refer to them as *Pseudomonas* sp. unknown, since the characterization is incomplete.

*“P. gingeri”* is an invalidly named species documented to produce ginger-colored superficial lesions. It is the only known causative agent of “ginger blotch” (Wong et al., 1982; Wells et al., 1996). Ginger blotch pathogens do not produce tolaasin (Lee et al., 2002) and their symptom development and epidemiology are poorly understood (Fletcher and Gaze, 2007). *“P. gingeri”* is phylogenetically closest to *P. agarici*, better known as the drippy-gill pathogen of *A. bisporus* (Young, 1970). In phylogeny, brown blotch pathogens are more closely related to each other than ginger blotch pathogens, which form separate clusters in phylogenetic trees (Godfrey et al., 2001; van der Wolf et al., 2016; unpublished results).

Bacterial blotch pathogens are believed to be endemic to the peat component of the casing soil, albeit at low densities. Once infected, secondary infection via insects, water splashing, mushroom pickers, and mechanized harvesters is quick (Wong and Preece, 1980). Given the mesophilic and humid conditions required for mushroom cultivation, pathogen densities are soon enriched in the mushroom beds (Wong et al., 1982; Godfrey, 2003). Limited management strategies exist for chemical, environmental, or biological control of blotch diseases (Godfrey, 2003; Fletcher and Gaze, 2007; Navarro et al., 2018; Osdaghi et al., 2019). Early and efficient detection of the pathogens is hence critical to predict and prevent blotch outbreaks.

For *P. tolaasii*, identification was formerly performed by a reaction between colonies of *P. tolaasii* and “*P. reactans”* in agar plates, referred to as the white line inducing principle (WLIP) (Wong and Preece, 1979; Goor et al., 1986; Han et al., 1992; Wells et al., 1996; Lloyd-Jones et al., 2005). However, closely related blotch-causing bacteria, such as *P. constantinii*, can also induce the white line precipitate against “*P. reactans”* (Munsch and Alatossava, 2002). WLIP has also been observed in isolates from the species complexes of *P. fluorescens* and *P. putida* (Rokni-Zadeh et al., 2012). Plating and phenotypic methods are thus unspecific for identification of *P. tolaasii* infection.

Recent advances allow qualitative detection of *P. tolaasii* using traditional and nested PCR methods (Lee et al., 2002). However, for other blotch pathogens like *“P. gingeri*,*”* even qualitative detection methods do not yet exist. There is a need for pathogen-specific quantitative diagnostic assays to track and quantify pathogen populations during the mushroom cultivation cycle and post-harvest chain. Identification of the pathogen, and knowledge of its population dynamics is essential to optimize early measures toward the prevention of blotch outbreaks.

Specific and sensitive molecular detection methods for blotch pathogens will help to solve current inconsistencies in symptom diversity and nomenclature of blotch-causing organisms. Quantitative detection methods will enable fundamental insights into pathogen population structures in the mushroom beds and on the caps, allowing study of the microbial ecology of the pathogens during the mushroom cropping process. The assays can also be used to monitor potential contamination of raw materials such as casing, compost (substrate), spawn (inoculated mycelium) water, and environments used for mushroom cultivation.

DNA-based amplification methods have gained wider acceptance and reliability than microscopy, phenotypic, serological techniques due to, its universal presence in cells of all kinds, the flexibility with respect to specificity, the high diagnostic sensitivity and the suitability for high-throughput testing (Schaad and Frederick, 2002; Ward et al., 2004). Formerly time-consuming PCR assays have now been replaced with Taqman^TM^-qPCRs, that not only quantify targets in real-time, but also increase the specificity of detection significantly (Mullis and Faloona, 1989; Mullis, 1990). The use of hydrolysis probes for measurement of sequence-specific amplification also allows detection of multiple targets in one reaction (Elnifro et al., 2000; Liu et al., 2013).

In this study we develop Taqman^TM^-qPCR assays diagnostic assays against three causative agents of bacterial blotch, that are currently causing severe disease outbreaks in Western Europe, *“P. gingeri,” P. tolaasii* and a not fully characterized *Pseudomonas* species, taxonomically related to *P. edaphica* and *P. salomonii*, that was formerly identified as *P. tolaasii*. We designed the assays for rapid and specific quantification of these blotch pathogens, at low population densities in a variety of environmental samples. The development of the assays and validation of their analytical performance, diagnostic performance, limitations, and potential applications are further elaborated.

## Materials and Methods

### Reference Strains

Reference strains for assay development were selected from a previous molecular characterization of blotch pathogens in which whole genomes were sequenced from 30 blotch-associated *Pseudomonas* (van der Wolf et al., 2016). Reference strains for *“P. gingeri*,*”* include LMG 5327^*T*^, LMG 5328, IPO 3754, IPO 3777, IPO 3776, IPO 3769, IPO 3757, IPO 2767, and IPO 3756. Reference strains for *P. tolaasii* include LMG 2342^*T*^, ATCC 51310, and ATCC 51309. Reference strains for the unknown brown-blotch causing *Pseudomonas* include LMG 2343 (formerly identified as *P. tolaasii*) and IPO 3765. Reference strains for each of the pathogens originate from symptomatic mushroom tissue and have been shown to demonstrate pathogenic behavior on fresh mushroom caps.

### Assay Development

Target regions against “*P. gingeri*” and the unknown *Pseudomonas* sp. were selected from Multi-Locus Sequence Alignment (MLSA) on whole genome sequences of *Pseudomonas* isolated from symptomatic mushrooms (van der Wolf et al., 2016; unpublished results). Reads from reference strains of each pathogen were aligned, and the consensus sequence was divided into 500 bp sequence blocks. Blocks that mapped to the pathogens of interest, but did not map to strains from any other outgroups, and had no single nucleotide polymorphisms (SNPs) were selected as the target regions. The sequence blocks were checked for specificity with NCBI-BLAST. Target regions against *P. tolaasii* were selected on the “tolaasin” gene fragment (accession numbers AY291584, AY228241, U16024, and AF291753) from LMG 2342^*T*^ and other isolates. Target regions were checked for homology to other bacterial species, in particular to taxonomically related pseudomonads via NCBI-BLAST. Assay development was done for six target regions that were specific to target *Pseudomonas* sp.

For each pathogen, two amplicons, with length between 80 and 120 bp, were designed on different loci within their respective target regions. TaqMan^TM^ primers and probes were designed using the PrimerQuest web-tool (Integrated DNA Technologies), or Primer Express (Applied Biosystems) with the oligo size between 18 and 25 bp (Table 1). The amplicons are described in Supplementary Table S1. All amplicons were subjected to BLAST searches against the *Pseudomonas* database^1^ to check for degeneracy. The specificity of the primers and probes was also checked via NCBI-BLAST. In total, six assays were developed against “*P. gingeri” (Pg2, Pg6), P. tolaasii (Pt1, Pt2)*, and the unknown *Pseudomonas* sp. *(Pu4, Pu10)*. The assays were multiplexed to also detect *Xanthomonas campestris* pv *campestris* (Xcc) (Köhl et al., 2011) and the *Pseudomonas* genus (Pp) (Lloyd-Jones et al., 2005).

**TABLE 1:** Description of TaqMan^TM^ primers, probes, amplicon size, and reporter dyes, for quantifying *“P. gingeri,” P. tolaasii*, an unknown *Pseudomonas* species, *X. campestris* pv. *campestris*, and the generic *Pseudomonas* population, abbreviated as Pg, Pt, and Pu, Xcc, and Pp, respectively.

Assays	Forward primer (5′–3′)	Reverse primer (5′–3′)	Probe (5′–3′)	Reporter dye
**Pathogen**				
Pg2	CACCGGACCGATGAAGG	CAGTGACTCGGGCTTGC	TCGGCGAAAGCCGTCTGATCACCGT	FAM
Pg6	TCATCCATGCAGTCGGAAAG	ACGCTGAACGCTCACATT	CAGCGACTTCACGACAGCGAACAC	FAM
Pt1	TGTTGTGCGCCTCGTTTTTA	AATGCGAGGGTCACTTTGGT	CCGCCGCACAGGCTCAGGA	FAM
Pt2	AGGCCGAAGGGCAAGGT	TGTCAGCGAGCAGGAGCAT	TGTCGATATCCCCGAGCAACTCGC	FAM
Pu4	GCAGATTGTCGCGTATTCC	ACCTGGCTGACGCCCGCTGC	ACGGTTTACGCGCCAATG	FAM
Pu10	ATGTTGATCACCTCGCCTTC	CGGGTGGAGAAGATTGCTTT	TTACGCTGTAGCGGGCAT	FAM
**Extraction Control**				
Xcc^a^	GTGCATAGGCCACGATGTTG	CGGATGCAGAGCGTCTTACA	CAAGCGATGTACTGCGGCCGTG	HEX
***Pseudomonas* Population**				
Pp^b^	GGGTGGTGGAATTTCCTGTGT	TTCCTTGTGGTCACCGCTTC	GTGAAATGCGTAGATATAG	ATT0 550

*^*a*^Köhl et al. (2011). ^*b*^Lloyd-Jones et al. (2005).*

### Reaction Conditions

The detection of bacterial DNA in varied samples was performed using TaqMan^TM^ probe technology, on a QuantStudio^TM^ 12K Flex from Applied Biosystems (Thermo Fisher Scientific, United States). Per reaction, the PCR mix included 0.25X ROX dye II, 1X TaKaRa^TM^ Premix Taq^TM^ DNA Polymerase (Takara Bio), 300 nM forward and reverse primers from all three targets, and 100 nM of FAM, VIC and ATTO labeled probes with NFQ-MGB quencher for target pathogens, *X. campestris* pv *campestris* and generic *Pseudomonas*, respectively. The total volume was made up with DNAse and RNase free water to 12.5 μl per reaction. Activation of Taq-polymerase was done at 95^°^C for 2 min The qPCR amplification conditions consisted of denaturation for 15 s at 95^°^C and extension for 60 s at 60^°^C, for 40 cycles. A Threshold cycle (C_T_) value lower than 35 was considered a positive reaction. qPCR reactions were performed with 1 ng of target DNA, in duplicates.

### Positive and Negative Controls

For determining the limits of detection, several types of positive controls were tested. The first positive controls consisted of gBlocks^®^ (Integrated DNA Technologies, United States). These synthetic oligonucleotides contain concatenated sequences of all amplicons to be tested within a multiplexed assay. gBlocks^®^ were designed per pathogen, for both target amplicons along with *X. campestris* pv *campestris* and generic *Pseudomonas* amplicons interspaced with some extra flanking nucleotides from the original gene sequence. The gBlock sequences are described in Supplementary Table S1. A serial dilution of the gBlocks^®^ from 10^8^ to 0 copies of target DNA was used to plot the calibration curves. Controls for water samples consisted of sterile water spiked with a pathogen suspension (in Ringers solution) to obtain a 10-fold serial dilution from 10^8^ to 0 cfu/ml. Controls for casing soil samples consisted of pure soil DNA, extracted from soil samples spiked with serially diluted pathogen suspension from 10^8^ cfu/g to a final concentration of 0 cfu/g of soil. All controls were spiked with three pathogens together. The negative control was DNAse and RNase free water.

### Sample Processing and DNA Extraction

Bacterial isolates were grown on King’s B or Trypic Soy agar for 24 h at 25^°^C before DNA extraction. The entire panel of bacterial strains are described in Supplementary Table S2. Total DNA was extracted from 200 mg of bacterial colonies scrapped from a plate for each isolate using Wizard Magnetic DNA Purification System for Food (Promega, United States) according to the manufacturer’s protocol, including the DNase-free RNAse treatment. All the DNA for the testing panel was quantified fluorometrically using a Quant-iT PicoGreen dsDNA assay Kit (Thermo Fisher Scientific, United States) on the Infinite M200 PRO microplate reader (Tecon, Switzerland).

Assays were also evaluated by testing a variety of environmental samples. Bacterial suspensions were prepared from pure cultures in Ringers solution, and serially diluted from an upper concentration of 10^8^ cfu/ml (OD_280_ = 0.1). Biopsies from symptomatic tissue were weighed, homogenized in 1 ml of Ringers solution, and the extract was filtered using BIOREBA bags (Bioreba, Switzerland). For water samples, 100 ml of tap-water was centrifuged at 9,000 g for 15 min at 4^°^C. The supernatant was discarded, and the pellet re-suspended in 1 ml of sterile DNAse and RNAse free water. The bacterial suspensions, tissue extracts and water samples were centrifuged at 11,200 g, then heated to 100^°^C for 10 min, and 1 μl of the supernatant was used for qPCR.

For casing soil samples received from mushroom farms, 250 mg of sampled material was processed for a semi-automated DNA isolation. The PowerMag Soil DNA Isolation kit from MoBio Technologies (Qiagen) was used to perform a total DNA extraction, eluted into 100 μl of Tris-EDTA buffer, using a Kingfisher Flex (Thermo Fisher Scientific) according to manufacturer’s protocol. 1 μl of the resultant DNA (concentration as extracted) was used for qPCR.

For diagnostic tests, 10^5^ cfu of *X. campestris* pv. *campestris* in 0.01 M phosphate buffer saline was added to water, mushrooms and soil samples before DNA extraction, to allow for relative quantification, by controlling for loss of bacterial DNA during sample processing.

### Data Analyses

Data analyses was performed on RStudio with R version 3.4.0 (R Core Team, 2013). Threshold cycle (C_T_) values from TaqMan^TM^ qPCR were imported from the QuantStudio^TM^ 12K Flex Real-Time PCR Software (Thermo Fisher Scientific, United States). When the C_T_ values were plotted against the copies of template DNA, amplicon efficiency was calculated from the slope of the calibration curve, as 10^–1/^*^*slope*^*−1 (Kubista et al., 2006). Pathogen populations in the samples were quantified relative to known concentrations of *X. campestris* pv. *campestris* by the 2^–ΔΔCT^ method (Livak and Schmittgen, 2001), as a fold change ratio.

## Results

### Development of Real-Time TaqManTM PCRs

**FIGURE 1:**
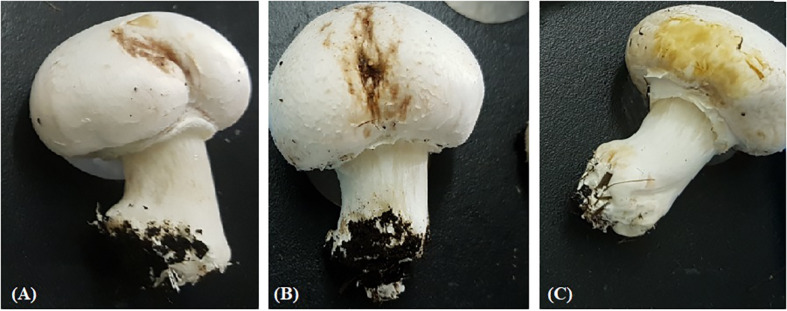
Symptomatic mushroom tissue naturally infected with bacterial blotch pathogen, **(A)** *P. tolaasii* (LMG2342), **(B)** unknown *Pseudomonas* species formerly identified as *P. tolaasii* (IPO3765) and **(C)** “*P. gingeri*” (IPO3777).

In this study, six TaqMan^TM^ qPCR assays were designed, to detect two different loci for each of the three blotch pathogens (Figure 1). Each target-pathogen assay was multiplexed with two additional assays that quantify *Xanthomonas campestris* pv. *campestris* (Xcc) (Köhl et al., 2011) as an extraction control, and the genus *Pseudomonas* (Lloyd-Jones et al., 2005) for the total *Pseudomonad* population (Pp). In total, six triplex assays were developed; two for “*P. gingeri*,*”* (i) Pg2-Pp-Xcc (ii) Pg6-Pp-Xcc; two for *P. tolaasii*, (i) Pt1-Pp-Xcc and (ii) Pt2-Pp-Xcc; and two for the unknown *Pseudomonas* sp., formerly classified as *P. tolaasii*, (i) Pu4-Pp-Xcc (ii) Pu10-Pp-Xcc.

Assay Pt1 and Pt2 amplify two fragments of the tolaasin gene (AF291753, AY291584). Assay Pg2 amplify the LysR transcriptional regulator gene fragment (PNQ94452) whereas assay Pg6 amplifies a hypothetical protein fragment (PNQ88072). Assays Pu4 and Pu10 amplify two unknown gene fragments. The Xcc assays also amplifies a hypothetical protein fragment (QCX70676). The Pp assay amplifies the 16S gene fragment (MK294319). In the reference genome sequences all amplicon targets are single copy. The sequences of primers and probes for the triplex assays are described in Table 1. The amplicons sequences are described in Supplementary Table S1. *In silico* homology of amplicons to taxonomically-related *Pseudomonas* sp. using NCBI-BLAST, is described in Supplementary Table S2.

### Analytical Performance

**FIGURE 2:**
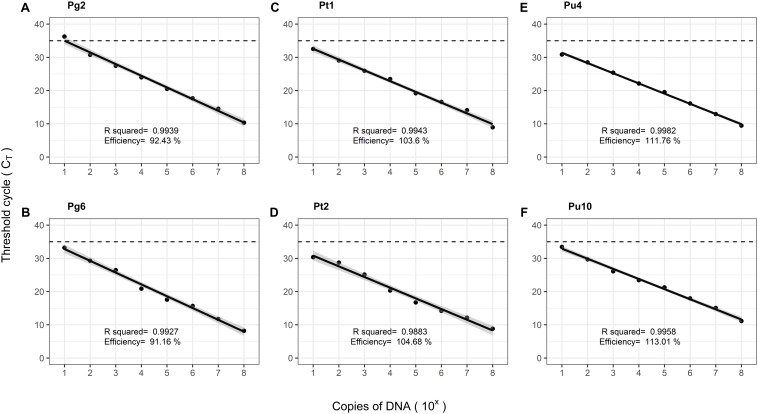
Calibration curves of multiplexed target assays **(A)** Pg2, **(B)** Pg6, **(C)** Pt1, **(D)** Pt2, **(E)** Pu4, and **(F)** Pu10, with amplification efficiencies and adjusted *R*^2^-values, when tested against positive controls comprising of serially diluted gBlocks^®^. The total copies of target DNA and the C_T_ values are plotted against the x and y axis, respectively. The gray bands indicate a confidence interval of 99%. And the dotted line indicates the cut-off value, C_T_ = 35.

**FIGURE 3:**
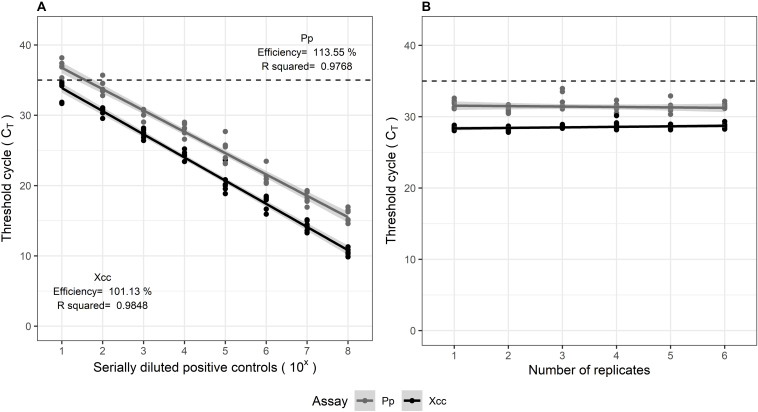
**(A)** Inter-assay (reproducibility) and **(B)** Intra-assay (repeatability) variation in multiplexed Xcc and Pp assays, tested on positive controls comprising of serially diluted gBlocks^®^. Both were tested in replicates of six.

The multiplexed assays for all target pathogens were tested against positive controls comprising of gBlocks^®^, in replicates of three. The TaqMan^TM^ assays were evaluated according to the Minimum Information for Publication of Quantitative Real-Time PCR Experiments (MIQE) guidelines (Bustin et al., 2009). The calibration curves showed good linearity, symbolized by the high correlation coefficients (*R*^2^ > 0.95). The amplification efficiencies (E) estimated from the slope of the curve, varied within 91.6–113%. The standard curves are illustrated in Figure 2. Delta Rn values (ΔRn) varied between 2.1 and 3.0 for the assays, suggesting a good magnitude of the amplification signal. The Xcc and Pp control assays both showed high precision, indicated by the low variation observed in qPCR results when tested in replicates of six, within and between experiments. Their repeatability (intra-assay variation) and reproducibility (inter-assay variation) is observed from the confidence intervals in Figure 3.

### Specificity and Sensitivity

A panel to test the diagnostic specificity, inclusivity and sensitivity was assembled from international (LMG, CFBP, ATCC, and NCPPB) and local culture collections. This panel included 18 bacterial blotch pathogens, 18 mushroom-associated *Pseudomonas*, 28 plant pathogenic *Pseudomonas*, and 41 other plant pathogenic bacteria (34 species from 14 genera). The assays were tested on 1 ng of total DNA from pure bacterial cultures. The true positive (TP), true negative (TN), false negative (FN), and false positive (FP) results are summarized in Tables 2, 3.

**TABLE 2:** Description of positive reactions (C_T_ < 35) of all six target assays, against the diagnostic bacterial panel.

Bacterial strains tested in panel for specificity and sensitivity	Positive results, where C_T_ < 35						
	Total	Pg2	Pg6	Pt1	Pt2	Pu4	Pu10
*“P. gingeri”*	8	8	8	0	0	0	0
*P. tolaasii*	8	0	0	8	8	0	0
P. unknown	2	0	0	0	0	2	2
Other mushroom-associated *pseudomonas*	16	1	1	0	0	1	0
Plant pathogenic *pseudomonas*	28	0	0	0	0	0	0
Other plant pathogenic bacteria	41	0	0	0	0	0	0

*Weak positive reactions, where C_T_ is close to the cut-off threshold (C_T_∼35), are marked in red.*

**TABLE 3:** Summary of diagnostic sensitivity and specificity of target assays.

Target assays	True positive	True negative	False positive	False negative	Diagnostic specificity	Diagnostic sensitivity
Pg2	8	94	1	0	0.99	1
Pg6	8	94	1	0	0.99	1
Pt1	8	95	0	0	1.00	1
Pt2	8	95	0	0	1.00	1
Pu4	2	100	1	0	0.99	1
Pu10	2	101	0	0	1.00	1

*Weak positive reactions, where C_T_ is close to the cut-off threshold (C_T_∼35), are marked in red.*

Diagnostic specificity of an assay is a measure of the negative samples that are correctly identified. It is described as TN/(TN + FP). The assays tested negative against the majority of other *Pseudomonas* strains and an assortment of plant-pathogenic bacteria. Very few cross reactions were observed with non-target strains, resulting in an overall specificity of 0.99, 0.99, 1, 1, 0.99, and 1 for Pg2, Pg6, Pt1, Pt2, Pu4, and Pu10, respectively. All non-specific amplifications were characterized by a very late detection of target DNA in one or both replicates, implying a weak positive reaction, where 35 > C_T_ > 40. Therefore C_T_ < 35 was considered an unambiguous positive result.

Diagnostic sensitivity of an assay is a measure of the positive samples that are correctly identified. It can be described as TP/(TP + FN). The six target assays, Pg2, Pg6, Put1, Pt2, Pu4, and Pu10, all tested positive for each of their respective reference strains, characterized by an early detection of DNA, where C_T_ < 25. No false negatives were detected, implying a high diagnostic sensitivity of 1. The mean C_T_ values from diagnostic specificity and sensitivity tests are described in Supplementary Table S3.

### Detection Thresholds

**FIGURE 4:**
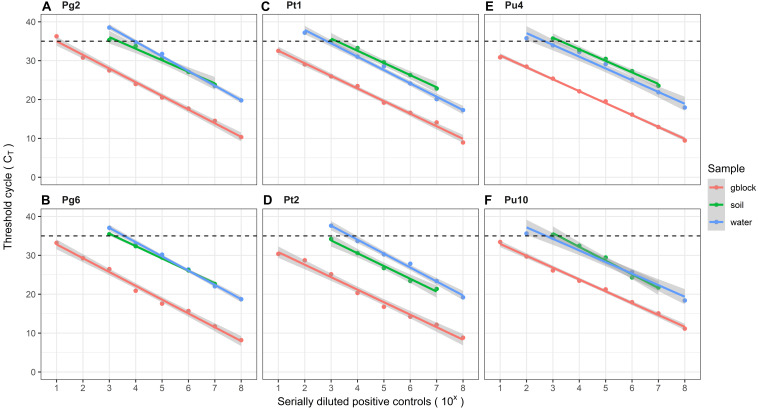
Mean detection thresholds of target-pathogen assays **(A)** Pg2, **(B)** Pg6, **(C)** Pt1, **(D)** Pt2, **(E)** Pu4, and **(F)** Pu10, in different sample types spiked with pathogen mixture, as described in the methods. gBlocks^®^, water samples and soil samples are depicted in red, green, and blue color, respectively.

The detection thresholds of all assays increase across target DNA (gBlocks^®^), spiked water and soil samples, indicated by the mean C_T_ values (Figure 4). The limit of detection (LOD), is described as the lowest concentration at which 95% of the positive samples are consistently detected, characterized by C_T_ < 35. When tested against serially diluted gBlocks^®^, assays Pg6, Pt1, Pt2, Pu4, Pu10, and Xcc had a LOD of 10 copies of target DNA. Assays Pg2 and Pp had a higher LOD of 100 copies of target DNA. For water samples, the LOD for assays Pt1, Pu4, and Pu10 was 10^3^ cfu/ml. Assays Pg2, P6, and Pt2 had a higher LOD of 10^4^ cfu/ml. For casing soil samples, assays Pt1 and Pt2 had the lowest LOD of 10^3^ cfu/g. Assays Pg2, Pg6, Pu4, and Pu10, had a higher LOD of 10^4^ cfu/g. The control assays, Xcc and Pp, gave consistent positive reactions at C_T_∼28 and C_T_∼30 for *X. campestris* pv *campestris* and the total *Pseudomonas* population in the soil, respectively.

### Diagnostic Application

Water, soil and symptomatic tissue from mushroom farms were tested to apply the newly designed assays. For primary detection, assays Pt1, Pg2, and Pu4 were employed, and the results were verified with the secondary assays, namely, Pt2, Pg6, and Pu4. Over the course of 10 independent batches in which samples were processed, diseased mushrooms, casing soil, peat, and tap water was sampled from mushroom farms across Netherlands and Belgium, and stored at -20^°^C until tested.

Diseased mushrooms were received from 50 sources varying in mushroom farm, soil, compost, cultivar, environmental conditions, and harvest cycle. Out of 95 symptomatic cap tissue tested, 78% of the samples were strongly positive (C_T_ < 25) for *“P. gingeri”* with assays Pg2 and Pg6, 42% of mushrooms tested positive for the unknown *Pseudomonas* species with the Pu10 and Pu4 assays, and 20% of the mushroom tested positive for *P. tolaasii* with Pt1 and Pt2 assays. Only 9.4% of the symptomatic samples tested negative against all assays. Several samples also showed a secondary infection with another pathogen, when multiple assays tested positive on the mushroom tissue. Immense variability exists between the pathogen densities on biopsies taken from mushroom caps, ranging from 4.8 × 10^2^ cfu to 7.9 × 10^6^ cfu. Pathogen density also varied with the severity of the disease symptoms.

Peat, before being mixed into casing soils in the mushroom farms, was received from 9 geographical sources. Out of 14 samples of peat tested, only 2 were positive for “*P. gingeri*” with assays Pg2 and Pg6, whereas 6 samples tested positive for the unknown *Pseudomonas* species with assays Pu4 and Pu10. These samples were not tested with assays Pt1 and Pt2 for *P. tolaasii*. Pathogen populations were consistently detected in the range from 1.2 × 10^5^ to 4.2 × 10^5^ cfu per gram. Co-occurrence of the pathogens was observed in only one peat sample, when it tested positive for multiple assays. Seven peat samples tested negative against all assays. Tap water from all samples tested unanimously negative for the blotch pathogens, characterized by C_T_ > 40.

## Discussion

### Overall Performance of Assays

All assays demonstrated high diagnostic specificity when tested against the bacterial panel, although occasionally weak false positives were detected, as observed in Supplementary Table S3. Multiple negative controls were also tested within the same setup, such as (a) no template control (b) sterile water control, (c) DNA extracted from a uninoculated plate of media. Contamination from positive controls during the qPCR, in reagents, or during DNA extraction is thus ruled out. In such samples, when DNA from pure bacterial cultures is used as template, due to very high DNA concentrations, template DNA can sometimes align with the fluorescent probe despite limited homology, leading to weak false positive reactions. Environmental samples, such as casing soil and water, contain much lower levels of pathogens, with the exception of symptomatic mushroom tissue. Hence these false positives are unlikely to occur when the diagnostic assays are applied during routine screening of raw materials used in mushroom farms. Furthermore the use of a C_T_ threshold value, determined from the assay validation data, allows interpretation of true positive and negative samples.

For diagnostic purposes, precise quantification of the very low density samples by qPCR is critical. All six target assays showed good linearity across the dilution range for their calibration curves, even at lower densities. The calibration curves of all assays showed similar slopes across the gBlocks^®^, water and soil samples, indicating that the qPCR efficiency is uninfluenced by sample type. The mean C_T_ values for the assays, at a given concentration of pathogen, increased from gBlocks^®^ and water to soils samples. This is potentially due to DNA loss during the extraction process, and can be addressed by improving DNA isolation methods for soil samples, which in turn can also reduce the presence of qPCR inhibitors such as humic and fulvic acids.

### Practical and Industrial Applicability

These assays can be successfully applied to identify and quantify pathogens on mushrooms, casing soils and peat sources at much lower densities than previously possible. Former thresholds of *P. tolaasii* density on mushroom caps were in the range from 7.7 × 10^4^ to 2 × 10^10^ cells per cap (Nair and Fahy, 1972; Nair and Bradley, 1980; Preece and Wong, 1982; Olivier et al., 1997). In the casing soil, pathogen densities were earlier reported in the magnitude of 10^7^–10^9^ cfu/g of soil (Nair and Fahy, 1972). For both caps and soils, 100-fold lower pathogen density thresholds were detected with the newly developed TaqMan^TM^ assays. This increased sensitivity, makes it now possible to screen raw materials used in mushroom cultivation, such as spawn, peat, compost and casing soil for presence of bacterial blotch pathogens.

Previously, the use of DNA-based molecular detection methods for routine diagnostics has been demonstrated for agricultural pathogens (Ward et al., 2004; Ophel-Keller et al., 2008; Bonants and te Witt, 2017). However, several considerations and limitations exist in the use of these assays to determine and provide insights into the “health status” of mushroom farms. The relationship between pathogen populations in the casing soil, and a disease outbreak is rather volatile. Blotch outbreaks depend on a variety of other factors also, which include environmental conditions, growing practices, cultivar type, source of compost and type of casing soil (Godfrey, 2003). The direct translation of pathogen population densities retrieved from these assays into prediction of an economic risk requires good knowledge and cautious interpretation of other disease indicators.

### Strengths and Shortcomings

Previously known diagnostic methods based on colony morphology and growth characteristics (Wong and Preece, 1979), microscopy (Preece and Wong, 1982; Soler-Rivas et al., 1999) and biochemical features (Goor et al., 1986), were time consuming and prone to false positive or -negative results. Conventional PCR assays (Kwon et al., 2000; Lee et al., 2002) require the use of gel electrophoresis for post amplification analyses, which make the assays less suitable for high throughput screening. Taqman^TM^-qPCR assays designed here, provide the opportunity for quantitative high-throughput detection of blotch pathogens at high precision, sensitivity and specificity in multiple environmental samples. Additionally, the assays ensure repeatability and accuracy in the diagnostic measurements.

A major limitation of these assays is that they only offer information on abundance of the pathogens, not its viability (Vincelli and Tisserat, 2008). The active fraction of pathogens cannot be calculated, as DNA-based assays cannot differentiate between dead and living cells (Wolffs et al., 2005). To quantify the fraction of viable cells, agents such as propidium monoazide may be used, that only penetrate dead cells with a compromised cell membrane and bind to DNA, after which the complexes can be to selectively removed prior to the TaqMan assay (Fittipaldi et al., 2012). The presence of viable cells by TaqMan assays may also be determined by enrichment of target cells in or on selective growth media (Schaad and Frederick, 2002) or reverse-transcriptase PCR to amplify mRNA instead (Kobayashi et al., 2009). However, these two methods do not quantify the fraction of viable cells accurately.

### Future Prospects

These assays present the first opportunity to quantify exact pathogen populations in environmental samples, and consequently study the microbial ecology and population dynamics of the blotch pathogens. They can be used in field-trials for testing the efficacy of biocontrol agents and disinfectants in mushroom beds. Based on population dynamics during the mushroom cropping cycle, they can be used to improve the dose and time of application. They can also be used to study the survival rates of these pathogens in the post-harvest supply chain, when introduced from the raw-materials, under varying storage conditions. Despite being limited to three different pathogens, these tools generate many fundamental insights for disease prevention, control and management.

For use of these assays as a monitoring system, an up-scaled sampling strategy needs to be explored for an industrial analysis. The number of samples that are representative of a batch, and the frequency of sampling that takes into account the effect of storage, seasonal variation, and bulk quantities, remain largely undetermined. Additionally, the use of an agent for removal of DNA from dead cells, could allow quantification of only the viable pathogen populations. Detailed information on the survival rates and population dynamics of the pathogen is essential to place quantitative information about pathogen densities into context. Finally, field trials are also required to understand the variation in the inoculum threshold of the soil for disease outbreaks, based on the type of casing soil, compost, cultivar, watering method and environmental conditions during production, etc.

## Conclusion

Six assays have been developed for quick and quantified detection of three aggressive bacterial blotch causing agents. The assays are highly specific and sensitive, and can be used to test for pathogen targets in different substrates associated with the mushroom cropping ecosystem, such as mushroom caps, water sources, peat, compost, and casing soils. They allow efficient diagnosis of secondary infections within the farms. Direct applications also lie in assessing the efficiency of applied disinfectants and bio-control agents. In case of generic disease symptoms, the assays can be used to verify the pathogen and apply specific disease control measures. Routine industrial application of these assays as a warning system require more insights into sampling strategies, pathogen population dynamics, and both abiotic and biotic disease indicators.

## Data Availability Statement

Publicly available datasets were analyzed in this study. This data can be found here: PNQ94452, PNQ88072, AF291753, AY291584, QCX70676, and MK294319.

## Author Contributions

MK and JH designed the diagnostic assays. TT, MK, JH, and MH performed the experiments. TT and JW wrote the first draft of the manuscript. JE critically reviewed the draft. All authors contributed to subsequent manuscript revision, read and approved the submitted version.

## Conflict of Interest

JE and JH were employed by Fera Science Limited. The remaining authors declare that the research was conducted in the absence of any commercial or financial relationships that could be construed as a potential conflict of interest.

## Footnotes

1 www.Pseudomonas.com

## Supplementary Material

TABLE S1 Description of target gene, accession number (if relevant), amplicon sequence, and gBlocks^®^ for each target assay.

TABLE S2 Description of homology of amplicon from target pathogens to 24 taxonomically-related *Pseudomonas* sp. *in silico* using NCBI-BLAST. It mentions the percentage identity and query cover for each reference strain. Blanks represent no significant similarity between reference and query sequence.

TABLE S3 Description of the strains that form the bacterial panel for evaluating the diagnostic specificity and sensitivity of the TaqMan^TM^ assays. It mentions the names and sources of reference strains from each blotch pathogen, other mushroom-associated *Pseudomonas*, plant pathogenic *Pseudomonas*, and other plant pathogens. It also describes their reaction (mean C_T_) against each assay.
